# Water Quality Characteristics and Seasonal Changes in Wastewater Treatment in the Southern Hebei Region by Branch

**DOI:** 10.3390/toxics13010008

**Published:** 2024-12-25

**Authors:** Chao Qu, Weiyuan Cao, Kun Dong, Dunqiu Wang, Yi Yao

**Affiliations:** 1 Guangxi Key Laboratory of Environmental Pollution Control Theory and Technology, Guilin University of Technology, Guilin 541006, China; qu109839@163.com (C.Q.); cwy12101115@163.com (W.C.); 2020005@glut.edu.cn (K.D.); wangdunqiu@sohu.com (D.W.); 2 Hebei Handan Ecological Environment Monitoring Center, Handan 056038, China; 3 Engineering Research Center of Watershed Protection and Green Development, Guilin University of Technology, Guilin 541006, China; 4 Modern Industry College of Ecology and Environmental Protection, Guilin University of Technology, Guilin 541006, China; 5 Collaborative Innovation Center for Water Pollution Control and Water Safety in Karst Area, Guilin University of Technology, Guilin 541006, China

**Keywords:** wastewater treatment plant, influent quality, temperature, treatment efficiency

## Abstract

This study analyzed three years of data (2021–2024) from three wastewater treatment plants (WWTPs), namely D, X, and T, in the main urban area of Handan, a typical city in the southern Hebei region, and investigated the influent characteristics and impact of temperature on these wastewater treatment facilities. With 90% assurance, the overall influent conditions of the three WWTPs in this region were normal. However, Plant T operated more effectively with slightly lower BOD_5_/COD_Cr_ (B/C), organic carbon/total phosphorus (C/TP), and organic carbon/total nitrogen (C/TN) ratios in the influent. Plant D consistently met the Level A standard, Plant X essentially reached the Level A standard, while Plant T attained the Level 2 standard prior to its upgrade. Following the upgrade, Plant T also steadily met the Level A standard. The effluent from all plants was relatively stable, primarily influenced by the influent characteristics and slightly influenced by temperature, but without having a noticeable impact on the effluent quality.

## 1. Introduction

Water pollution and scarcity are critical challenges in the development of northern arid cities. One avenue through which these issues are resolved is water pollution control together with water recycling [1,2]. In northern China, where water resources are particularly scarce, effective wastewater treatment is essential not only to maintain water quality but also to ensure the availability of reclaimed water for various uses such as agriculture, industrial processes, and even indirect potable reuse. The sustainable management of water resources in this region is crucial for supporting urban growth and mitigating the environmental impacts of the overextraction of groundwater, which can lead to land subsidence and the degradation of local ecosystems. Through stably operated wastewater treatment plants (WWTPs), water pollution can be controlled, water environmental quality can be improved, and stable-quality reclaimed water can be provided. This enhances the reuse efficiency of water resources, mitigates the overextraction of groundwater, and realizes the efficient and orderly utilization of urban water resources [3,4,5,6]. Moreover, the reuse of treated wastewater can play a major role in reducing the pressure on freshwater resources, contributing to the resilience of water supply systems in the face of climate change. This is particularly relevant in regions such as Hebei, where urbanization is rapidly increasing the demand for water, while climate change is exacerbating water scarcity.

Urban WWTPs are vital infrastructures that not only help to maintain water quality but also act as a critical component of the urban water cycle. Treated effluent can be used to recharge local aquifers, support river flows during dry seasons, and irrigate agricultural lands, thereby playing a multifaceted role in regional water management strategies. The multifunctional role of WWTPs highlights the need for their efficient and stable operation, particularly in areas where water resources are under severe stress. Hence, urban WWTPs are a crucial infrastructure in cities, representing the primary means of maintaining a well-regulated social water cycle both domestically and internationally [7]. The efficient and stable operation of WWTPs plays an important role in the efficient utilization of urban water resources. However, achieving this efficiency is not without its challenges. The treatment processes within WWTPs are highly dependent on various environmental factors, including temperature, pH, and the characteristics of the influent water, which can vary considerably owing to seasonal changes, industrial discharges, and urban runoff. These variations can affect biological processes that are central to wastewater treatment, leading to fluctuations in treatment efficiency and effluent quality. The toxic effects of different types of pollutants on wastewater treatment plants vary. BOD_5_ can reflect the concentration of organic matter in sewage. The BOD_5_ value of incoming and outgoing wastewater can indicate the concentration of organic matter in incoming and outgoing water, respectively; a large amount of organic matter into the biochemical tank will exceed the microbial processing capacity, resulting in the incomplete decomposition of organic matter and thus the water quality not meeting the standard. At the same time, too high COD_Cr_ will make the oxygen consumption increase dramatically and reduce the level of dissolved oxygen, affecting the activity of microorganisms. Ammonia nitrogen in water is mainly in the form of free ammonia (NH_3_) and ammonium ions (NH_4_^+^). Free ammonia is highly toxic to both aquatic organisms and humans. Although the direct toxicity of ammonia nitrogen to the human body is relatively small, under certain conditions (such as high temperature, high pH, etc.), the proportion of free ammonia will increase, thus enhancing toxicity. Total nitrogen and nitrate nitrogen in the human body has a toxic effect. Nitrite nitrogen can oxidize the body’s normal hemoglobin into methemoglobin, which loses the ability to transport oxygen, leading to tissue hypoxia.

The need to optimize WWTP operations in the face of these challenges has led to a growing interest in the development of smart water management systems. These systems utilize real-time monitoring, advanced data analytics, and automation to dynamically adjust treatment processes, ensuring consistent performance irrespective of varying conditions. By integrating such technologies, WWTPs can improve their operational resilience and reduce energy consumption and operational costs, contributing to the overall sustainability of urban water management. However, most WWTPs primarily employ biological treatment processes, centered on microbial activities that adhere to certain biological behavioral norms. The treatment efficiency is greatly affected by environmental conditions such as dissolved oxygen (DO), temperature, pH, and substrates (influent characteristics) [8,9]. Although effective under optimal conditions, these biological processes can be particularly sensitive to disturbances. For instance, sudden drops in temperature during winter can slow down microbial activity, leading to reduced treatment efficiency. Similarly, fluctuations in the organic load of the influent can overwhelm a system, causing a temporary deterioration in effluent quality. Addressing these challenges requires a deep understanding of the interactions between the environmental factors and biological processes within a treatment system. Consequently, temperature and influent characteristics are not only important factors affecting water treatment efficiency but are also fundamental for WWTP design, operational regulation management, and the future smart operation of water plants [10,11,12].

Urban wastewater characteristics are easily influenced by the regional environment, climatic conditions, economic development, lifestyle habits, and industry type, with noticeable monthly variations [13]. Moreover, northern cities experience substantial temperature differences across seasons, with varying average annual temperatures and both lowest and highest temperatures. This leads to a disparity between the actual influent quality and the design quality in some WWTPs, remarkably interfering with the operation and management of WWTPs and presenting substantial risks. Against this background, this study performed analyses based on three years of monitoring data from three WWTPs (employing three different processes) in Handan City. By considering the annual temperature differences across various seasons and evaluating the impact of temperature and substrates (influent characteristics) on the operation of these three WWTPs, this study analyzed the correlation between environmental factors and pollutants. This analysis aimed to provide a scientific basis for the efficient and stable operation of WWTPs, as well as the smart management of water plants.

## 2. Materials and Methods

### 2.1. Overview of WWTPs

The central urban area of Handan City hosts three urban WWTPs, named D, T, and X. The basic details are listed in Table 1. The drainage system in the main urban area of Handan adopts a separate system for stormwater and wastewater. Population equivalent (p.e.) and the total amount of organic pollutants in an industrial wastewater stream, expressed as the number of people polluted by domestic sewage, are shown in Table 1. Based on the wastewater discharge routes, the wastewater network system is divided into two major systems: the Eastern Wastewater System (comprising Plants D and T) and the Western Wastewater System (Plant X). The Eastern Wastewater System collects wastewater from areas east of the Beijing–Guangzhou railway, south of the Qin River, west of the expressway, and north of the Zhi Zhang River, covering a drainage area of 46.1 km^2^ with a planned service population of 730,000. The Western Wastewater System primarily collects wastewater from areas west of the Beijing–Guangzhou railway and is east of the Qin River, west of the Fu yang River, and south of the Northern Ring Road, covering a service area of 50 km^2^ with a population of 450,000 [14].

### 2.2. Process Flow

**Figure 1:**
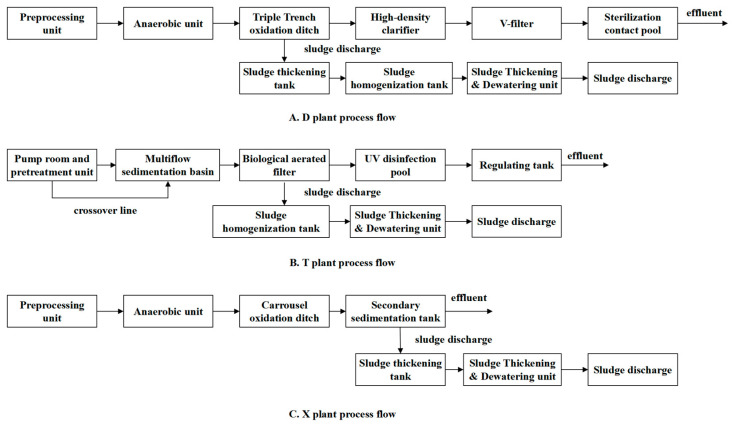
Operating process of the three wastewater treatment plants.

The three WWTPs in the central urban areas have been operating well in recent years, and their process flows are illustrated in Figure 1.

Plant D, with a design capacity of 160,000 m^3^/d, primarily employs a triple oxidation ditch + high-density clarification tank + V-type filter process. The triple oxidation ditch + high-density clarification tank + V-type filter process integrates aeration and sedimentation processes and has the characteristics of alternating operation in time sequence, and its operation cycle can be adjusted according to the different quality of treated water, thus making its operation more flexible and convenient. This kind of process is simple, and there is no need to set up primary and secondary sedimentation tanks and sludge reflux devices, which greatly reduces the infrastructure investment and operation cost of the oxidation ditch process and solves the disadvantage of having a large area occupied by an oxidation ditch, which happened in the past to a certain extent. The designed effluent quality meets the Level 1 standard of the “Integrated Wastewater Discharge Standard” (GB8978–1996) [15], with an actual operating capacity of 60,000 m^3^/d.

Plant T, designed with a capacity of 100,000 m^3^/d, adopts an aerated biological filter as its main process. An aerated biological filter has the advantages of high organic load, a small footprint (1/3 of common activated sludge method), low investment (saving 30%), no sludge expansion, high oxygen transfer efficiency, good effluent quality, etc. However, it has more stringent requirements for the SSs of influent water (generally SSs ≤ 100 mg/L, preferably SSs ≤ 60 mg/L), so the influent water needs to be pretreated. At the same time, due to the process of backwashing water, head loss is larger. The designed effluent quality conforms to the Level 1B standard of the “Urban Wastewater Treatment Plant Pollutant Discharge Standard” (GB18918–2002) [16], operating at a real capacity of 60,000 m^3^/d.

Plant X, also with a design scale of 200,000 m^3^/d, principally utilizes an improved Carrousel oxidation ditch process. In the improved Carrousel oxidation ditch process, the wastewater is operated by anaerobic, anoxic, and aerobic alternately, which has the effect of the simultaneous removal of organic matter, denitrogenation, and phosphorus removal. The process not only has the characteristics of A^2^O; it can also preserve the unique water flow characteristics of the oxidation ditch, which is conducive to the biological cohesion of activated sludge, and does not need to set up the primary sedimentation tank and sludge digestion tank, saving on investment. The designed effluent quality meets the Level 2 standard of the “Urban Wastewater Treatment Plant Pollutant Discharge Standard” (GB18918–2002), with an actual operating capacity of 85,000 m^3^/d [17].

The selection of specific processes for each plant was based on various factors, including local environmental regulations, economic considerations, and the specific characteristics of the influent wastewater. For example, the triple oxidation ditch process used in Plant D was selected because of its ability to handle high organic loads and its flexibility in adapting to varying flow rates, which is crucial in areas with considerable seasonal fluctuations in wastewater generation. The aerated biological filter in Plant T was selected for its compact design and energy efficiency, making it suitable for densely populated urban areas with limited space. To meet the urban water environment management requirements, Plants T and X underwent an upgrade and operation optimization at the end of 2022, elevating their effluent standards to Level 1A of the “Urban Wastewater Treatment Plant Pollutant Discharge Standard” (18918–2002).

### 2.3. Water Quality and Analytical Methods

Water quality was monitored once daily. The analytical methods primarily referred to the “Water and Wastewater Monitoring and Analysis Methods” (Fourth Edition) [16]. COD_Cr_ was determined using the potassium dichromate method (DR2800, HACH, Loveland, CO, USA); NH_4_^+^-N, TN, and total TP were measured using a continuous-flow injection analyzer (SAN++, SKALAR, Breda, The Netherlands); SSs were determined using the filtration gravimetric method; and pH and DO contents were measured using a multifunctional water quality analyzer (HQ43d, WTW, Munich, Germany) [18]. To ensure the accuracy and reliability of the data, all instruments were regularly calibrated according to the manufacturers’ guidelines, and quality control procedures were implemented throughout the sampling and analysis processes. Additionally, this study incorporated redundant sampling at selected intervals to assess the consistency of the data and identify potential anomalies. The collected data were further subjected to statistical analysis to determine the significance of the observed trends and correlations, providing a robust basis for subsequent analysis of the WWTPs’ performance.

## 3. Results and Discussion

### 3.1. Monitoring Organic Matter (Expressed as COD_Cr_ and BOD_5_)

**Figure 2:**
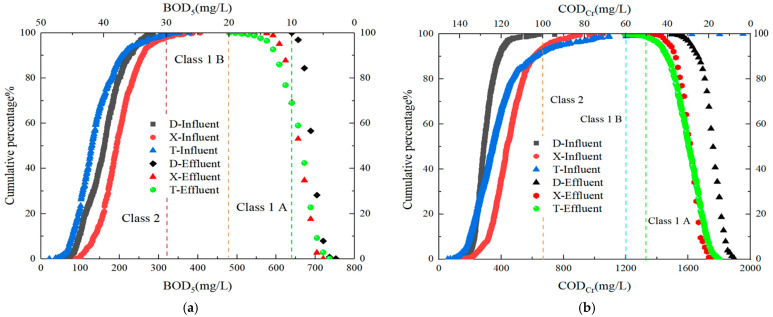

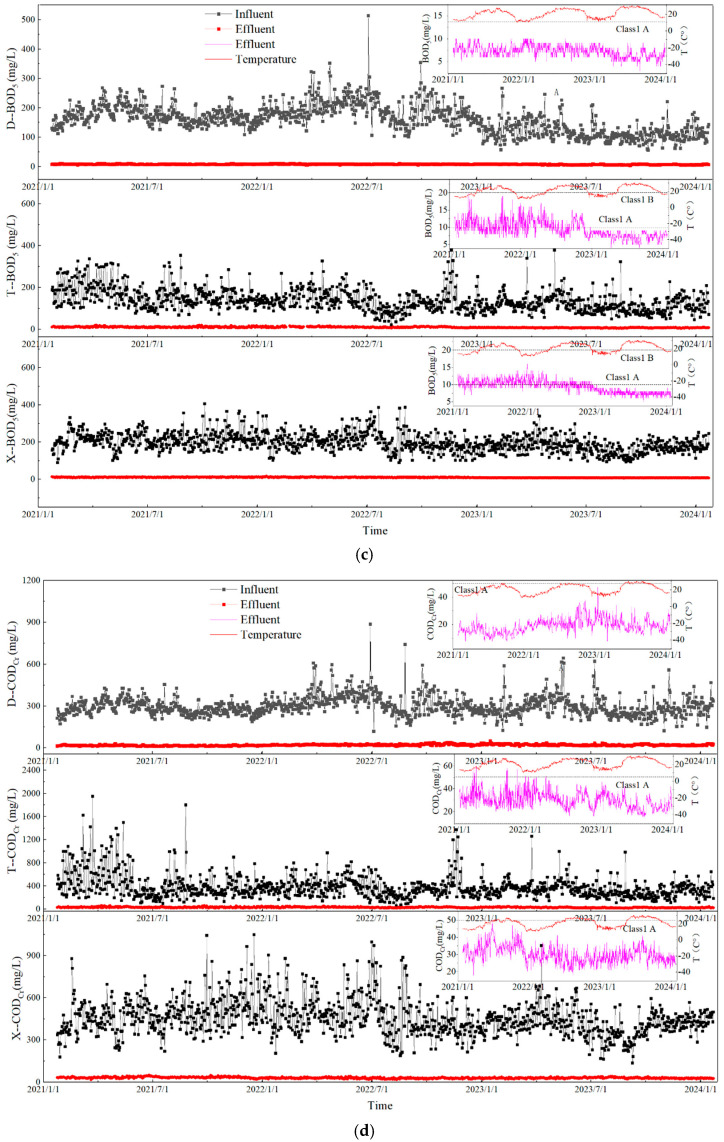

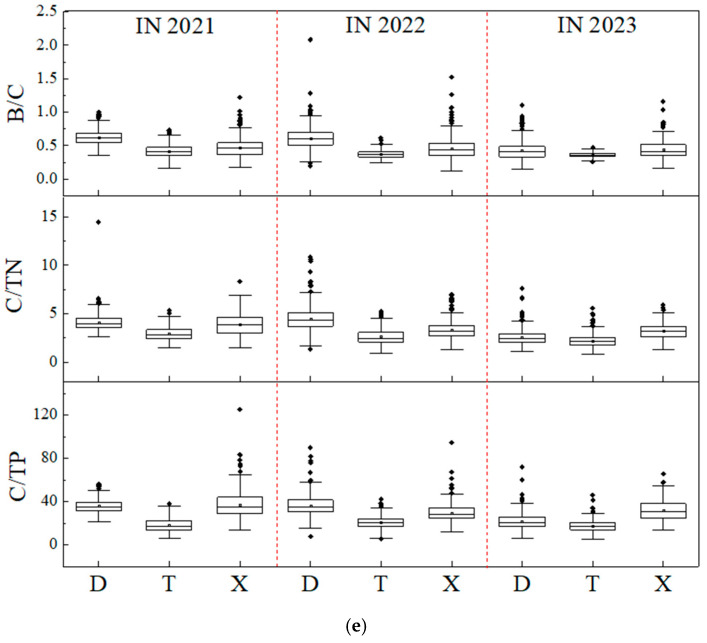
Analysis of influent and effluent characteristics in three wastewater treatment plants ((**a**) Cumulative probability and distribution characteristics of influent BOD_5_; (**b**) cumulative probability and distribution characteristics of influent COD_Cr_; (**c**) temporal variation in influent and effluent BOD_5_; (**d**) temporal variation in influent and effluent COD_Cr_; (**e**) characteristics of influent B/C, C/TN, and C/TP ratios).

The cumulative probability and distribution characteristics of influent BOD_5_ and COD_Cr_ for the three WWTPs in the main urban area of Handan City are shown in Figure 2. At a 90% assurance level, the concentration ranges of the influent BOD_5_ and COD_Cr_ for the three plants were 206–261 and 386–621 mg/L, respectively, among which Plant X had relatively high influent BOD_5_ and COD_Cr_, and Plant T had a relatively low B/C ratio (Figure 2e). The median in 2021 was greater than 0.4, the upper quartile (Q1) in 2022 exceeded 0.4, and the upper quartile (Q3) in 2023 was slightly less than 0.4, indicating the presence of hard-to-biodegrade pollutants in the influent [19].

The observed variations in BOD_5_ and COD_Cr_ across the three plants highlighted the influence of regional and seasonal factors on wastewater composition. For instance, the higher influent concentrations of Plant X suggested a greater presence of organic pollutants, which could be attributed to the industrial discharges or denser urban populations in its catchment area. These differences underscore the need for tailored treatment strategies that consider the specific characteristics of the influent in each plant. Additionally, the lower B/C ratio in Plant T indicates a relatively lower biodegradability of the organic matter present, which may require enhanced treatment processes, such as advanced oxidation or the addition of external carbon sources, to improve biodegradation efficiency.

This resulted in slightly inferior effluent quality from Plants X and T. The compliance rates for the Level A standard for effluent BOD_5_ in Plants D, T, and X were 95.7%, 68.4%, and 69.2%, respectively, whereas for the Level B standard, they were 100%, 98.1%, and 100%, respectively; for effluent COD_Cr_, the compliance rates for the Level A standard were 100%, 97.5%, and 100%, and for the Level B standard, they were 100%, 99.8%, and 100%, respectively. However, in terms of effluent stability, all three plants performed well and were relatively stable, with a minor influence from temperature.

This stability in effluent quality, despite variations in influent characteristics, suggested that the treatment processes were sufficiently robust to handle fluctuations. However, the lower compliance rates for BOD_5_ in Plants T and X, compared to that of Plant D, indicated that these plants may benefit from process optimization or additional treatment stages, particularly during periods of high organic loads. Furthermore, the role of operational parameters, such as sludge retention time (SRT) and aeration intensity, should be investigated to identify potential improvements that could enhance the overall treatment efficiency and effluent quality.

After 2023, the effluent BOD_5_ from Plants T and X decreased to a lower level, stabilizing at Level A or above, primarily because of the upgrade and operational optimization conducted in Plants T and X.

The positive impacts of these upgrades highlight the importance of continuous monitoring and the periodic enhancement of treatment facilities to meet evolving environmental standards.

### 3.2. NH_4_^+^-N and TN Variations

**Figure 3:**
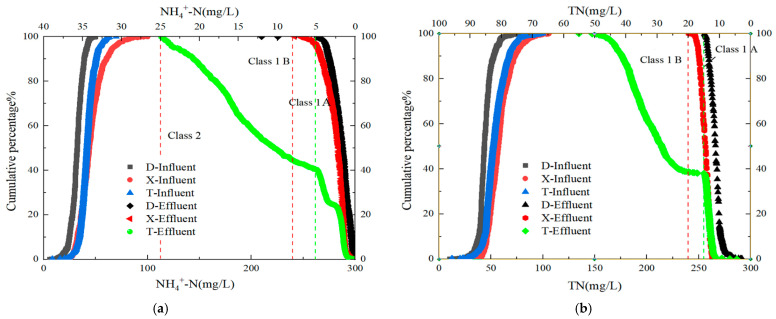

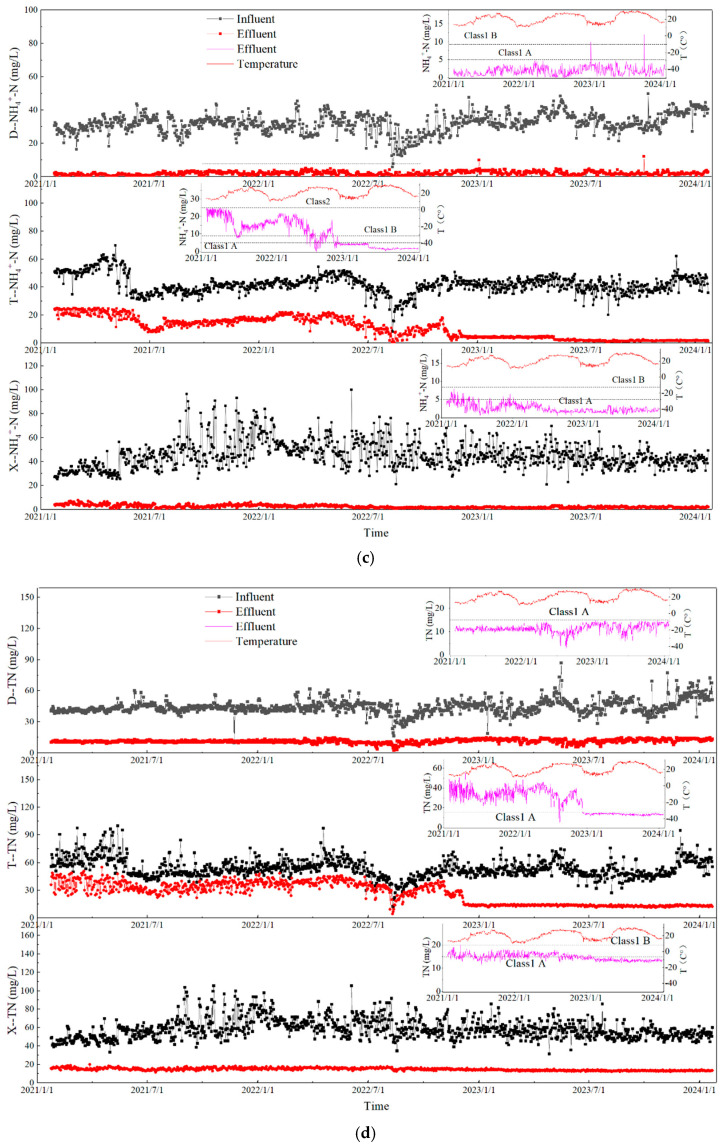
Analysis of influent and effluent characteristics in three wastewater treatment plants ((**a**) Cumulative probability and distribution characteristics of influent NH_4_^+^-N; (**b**) cumulative probability and distribution characteristics of influent TN; (**c**) temporal variation in influent and effluent NH_4_^+^-N; (**d**) temporal variation in influent and effluent TN).

The cumulative probabilities and distribution characteristics of influent NH_4_^+^-N and TN for the three WWTPs in the main urban area of Handan City are shown in Figure 3. At a 90% assurance level, the concentration ranges of influent NH_4_^+^-N and TN for the three plants were 16.4–33.2 and 32.6–54.9 mg/L, respectively. Plant X exhibited relatively high influent NH_4_^+^-N and TN levels, indicating a greater nitrogen load than that of the other plants. This higher nitrogen load in the influent of Plant X could be attributed to the higher proportion of industrial discharge or areas with a high population density within its catchment. These factors contribute to the complexity of nitrogen removal processes, particularly during periods of low temperature, where nitrification can be considerably inhibited.

The effluent compliance rates for the Level A standard for NH_4_^+^-N in Plants D, T, and X were 96.2%, 84.6%, and 78.4%, respectively, whereas for the Level B standard, they were 100%, 98.6%, and 100%, respectively; for effluent TN, the compliance rates for the Level A standard were 95.3%, 79.2%, and 76.1%, and for the Level B standard, they were 100%, 96.7%, and 100%, respectively. These compliance rates suggested that, while all three plants were generally effective in nitrogen removal, there were notable differences in their performance, particularly for Plant X, which faced greater challenges in meeting the stricter Level A standard. The lower compliance rates for TN in Plant X may indicate the need for enhanced denitrification processes, especially during colder months when the biological nitrogen removal efficiency typically declines.

Seasonal variations have a remarkable impact on the performance of WWTPs, particularly on nitrogen removal. During the winter months, nitrification was inhibited because of lower temperatures, leading to higher effluent NH_4_^+^-N concentrations. The operational data from Plants T and X showed a marked increase in effluent NH_4_^+^-N and TN levels during the colder months, with compliance rates dropping by as much as 15% compared to those during the summer months. This seasonal impact highlights the need for adaptive management strategies, such as adjusting the SRT or implementing supplemental heating in bioreactors to maintain optimal conditions for nitrification during winter. Additionally, integrating advanced nitrogen removal technologies, such as anammox or moving bed biofilm reactors, could enhance nitrogen removal efficiency, particularly in cold climates.

In contrast, Plant D, which employs a triple oxidation ditch process, showed a relatively stable performance across all seasons, with only minor fluctuations in the effluent NH_4_^+^-N and TN concentrations. This stability could be attributed to the ability of the process to maintain a consistent environment for nitrifying bacteria, even under varying temperature conditions. The design and operational flexibility of the oxidation ditch allowed for better control of aeration and mixing, which is crucial for effective nitrogen removal. Therefore, Plant D’s approach could serve as a model for optimizing nitrogen removal processes in other WWTPs facing similar challenges.

Overall, the data indicate that, although all three plants are capable of achieving the required effluent standards for NH_4_^+^-N and TN, there is room for improvement, particularly in Plants T and X. Further research should focus on optimizing the operational parameters of these plants, such as aeration strategies, carbon source dosing for denitrification, and the potential use of bioaugmentation to enhance microbial activity. Such improvements could help achieve more consistent nitrogen removal and better compliance with stringent effluent standards, even under challenging conditions.

### 3.3. TP and Suspended Solid (SS) Variations

**Figure 4:**
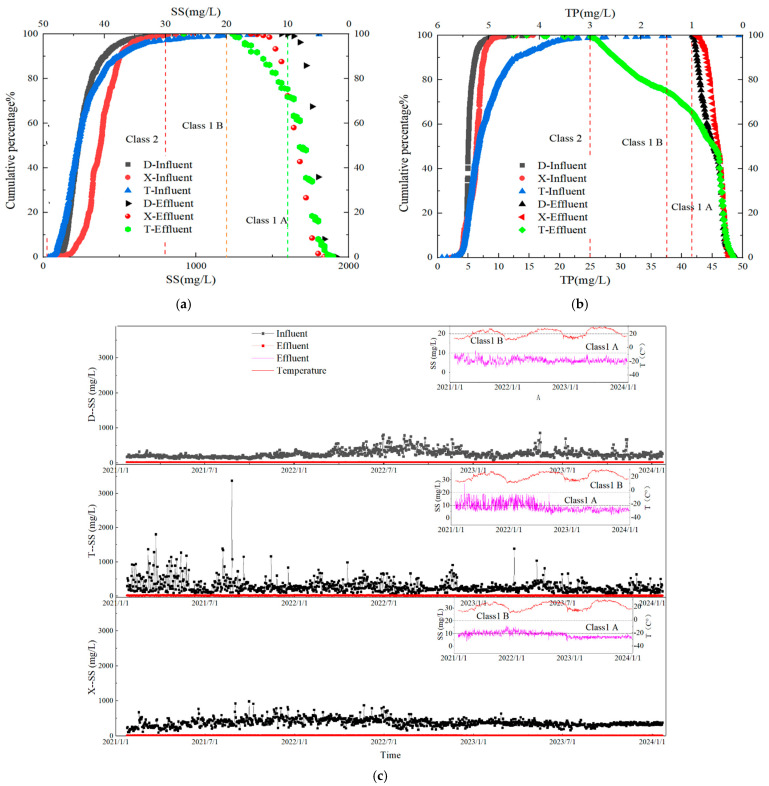

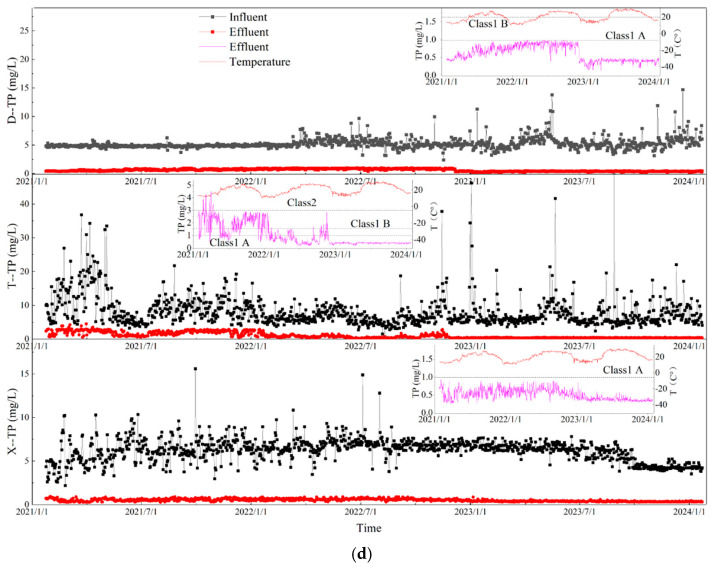
Cumulative probability and distribution characteristics of influent SSs and TP in three wastewater treatment plants in Handan’s main urban area ((**a**) Cumulative probability and distribution characteristics of influent SSs; (**b**) cumulative probability and distribution characteristics of influent TP; (**c**) temporal variation in influent and effluent SSs; (**d**) temporal variation in influent and effluent TP).

The cumulative probabilities and distribution characteristics of the influent concentration of TP and Suspended Solids (SSs) for the three WWTPs in the main urban area of Handan City are shown in Figure 4. At a 90% assurance level, the concentration ranges of influent TP and SSs for the three plants were 3.4–6.9 and 178–287 mg/L, respectively. Plant D exhibited relatively high influent TP concentrations, indicating a significant phosphorus load, whereas Plant X had higher influent SS levels, suggesting a higher presence of SSs. The higher TP concentration in the influent of Plant D could be linked to industrial activities within its catchment area that discharge phosphorus-rich wastewater. The elevated SS levels in Plant X could be attributed to stormwater runoff or unregulated industrial discharge that introduce particulate matter into the sewer system. These factors highlight the importance of source control measures and pretreatment requirements for industries to reduce the pollutant load entering WWTPs.

WWTPs typically employ biological phosphorus removal processes [19], with the phosphorus removal capability being positively correlated with the energy gained by phosphorus-accumulating organisms (PAOs) and the amount of phosphorus removed from the system. Hence, the phosphorus removal capability was significantly affected by the influent C/TP ratio. Lu et al. [20] found that a better phosphorus removal performance could be achieved when the C/TP ratio is higher than 20. The effluent compliance rates for the Level A standard for TP in Plants D, T, and X were 92.1%, 85.3%, and 81.7%, respectively, whereas for the Level B standard, they were 100%, 98.9%, and 99.5%, respectively. For effluent SSs, the compliance rates for the Level A standard were 94.6%, 89.2%, and 86.8%, respectively, and for the Level B standard, they were 100%, 97.7%, and 98.2%, respectively. These results indicated that while the plants generally performed well in removing phosphorus and SSs, Plant X consistently showed lower compliance rates, particularly for SSs. This may point to limitations in the removal processes for solids, such as sedimentation or filtration, which may require optimization or the introduction of advanced treatment stages, such as membrane filtration or enhanced coagulation, to achieve better removal efficiencies.

Seasonal variations also significantly affected the performance of the WWTPs, particularly for phosphorus removal. During colder months, the biological uptake of phosphorus was less efficient owing to reduced microbial activity, leading to higher effluent TP concentrations. The operational data from Plants T and X showed increased effluent TP during winter, with compliance rates dropping by 10–15% compared to those in the warmer months. This seasonal decline in the phosphorus removal efficiency underscores the need for adaptive operational strategies, such as adjusting chemical dosing for phosphorus precipitation or enhancing biological phosphorus removal through process modifications such as anaerobic/anoxic phase extension. Additionally, integrating side-stream treatment processes, such as struvite precipitation, could help manage phosphorus loads more effectively during periods of low biological activity.

In contrast, Plant D, which employed a triple oxidation ditch process combined with a high-density clarification tank, demonstrated more stable performance across all seasons, with only minor fluctuations in the effluent TP and SS concentrations. The stability of Plant D could be attributed to its robust process design, which allowed for the effective separation of solid and phosphorus removal, even under varying environmental conditions. In particular, the high-density clarification tank played a crucial role in enhancing solid–liquid separation, thereby improving the overall treatment efficiency and effluent quality. This design could serve as a reference for other WWTPs aiming to improve their performance, particularly in regions with considerable seasonal variations. Overall, although the data suggest that the three WWTPs were generally effective in achieving the required effluent standards for TP and SSs, there is potential for further optimization.

### 3.4. Correlation Analysis

**Figure 5:**
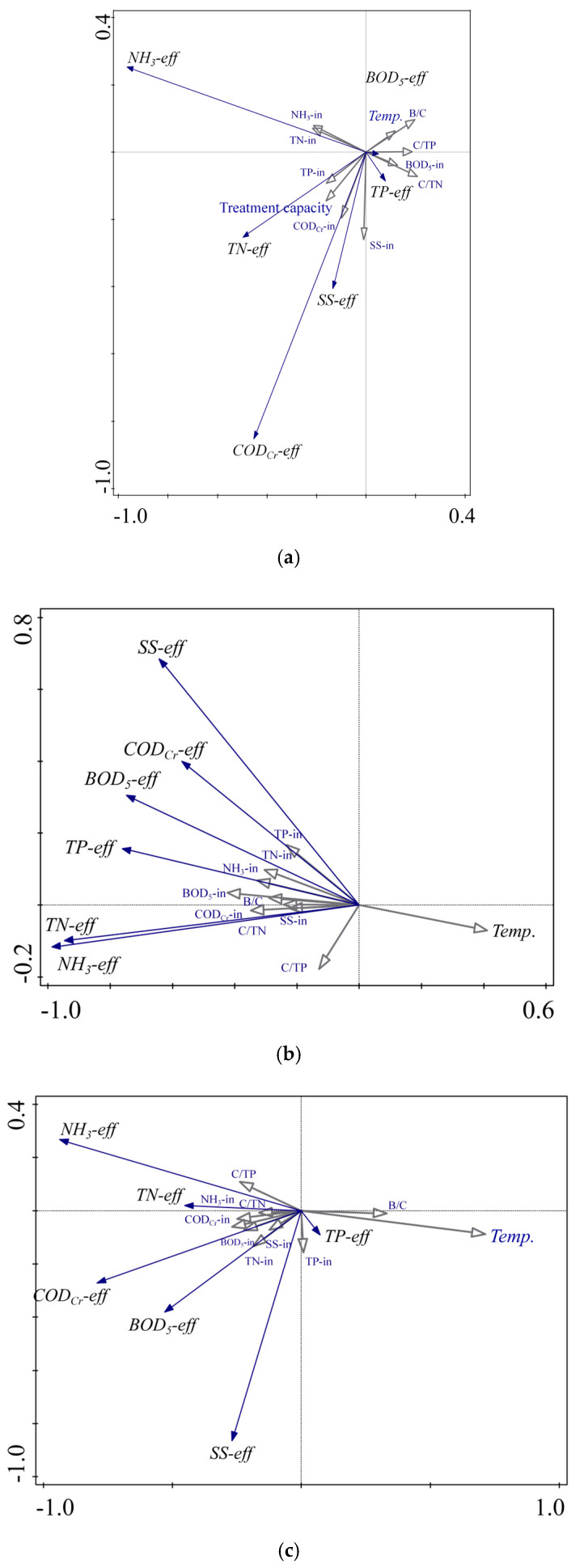

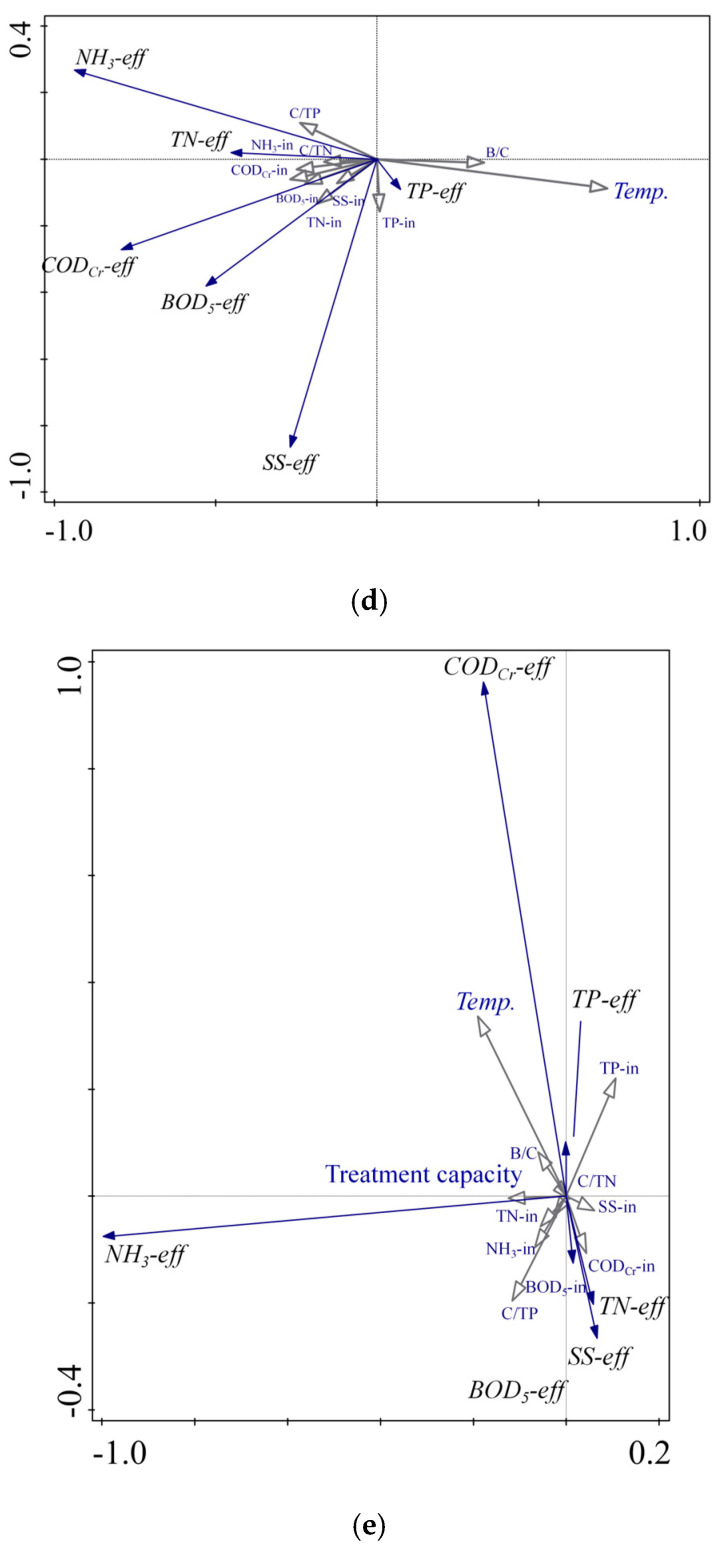
Correlation analysis of water in and out of three sewage treatment plants ((**a**) Plant D 2021–2024; (**b**) Plant X 2021–2024; (**c**) Plant X 2023; (**d**) Plant T 2021–2024; (**e**) Plant T 2023).

Figure 5 illustrates the redundancy analysis results of the three-year data from WWTPs D, X, and T. Correlations between various influent and effluent parameters were analyzed to understand the interdependencies and potential effects of different factors on the overall treatment performance of the WWTPs. Correlation analysis utilized Pearson’s correlation coefficients, which offer insights into the linear relationships between the variables under investigation. This analysis is essential for identifying key operational parameters that require adjustment to optimize the treatment efficiency, particularly under fluctuating influent conditions due to seasonal or industrial variations.

Since the technical renovation of wastewater Plants X and T was completed at the end of 2022, the data for 2023 for both plants were analyzed separately. It can be seen that before and after the renovation, the influencing factors for the effluent quality of the two wastewater Plants X and T were significantly reduced. The results demonstrated a strong positive correlation between the influent BOD_5_ and COD_Cr_ concentrations, suggesting that these two parameters were closely related and primarily affected by similar sources of organic pollution. This connection underscores the importance of source control and pretreatment strategies in managing organic loading to ensure consistent treatment performance. Additionally, the strong correlation between BOD_5_ and COD_Cr_ in the influent emphasized the need for robust treatment processes capable of effectively managing high organic loads, particularly in areas with substantial industrial discharge.

The effluent NH_3_ and TN concentrations in each plant were primarily influenced by factors such as temperature, C/TN ratio, C/TP ratio, and the concentrations of NH_4_^+^-N and TN in the influent. This was likely due to the dual effects of microbial limitations and the competitive nature of PAOs [21,22]. The strong correlation between NH_4_^+^-N and TN in the effluent suggested that fluctuations in the ammonia concentration directly affected the nitrogen removal efficiency. This correlation underscores the need to optimize nitrification and denitrification processes to maintain stable TN removal, particularly during colder periods when microbial activity diminishes. Additionally, the moderate correlation between the influent pH and effluent ammonia concentration highlighted how deviations from the optimal pH range can impair nitrification, leading to incomplete ammonia oxidation and elevated effluent ammonia levels.

The analysis also revealed a moderately negative correlation between influent temperature and effluent TP concentration, indicating that lower temperatures may hinder phosphorus removal owing to the reduced microbial activity and slower reaction kinetics in biological processes. The significant impacts of the influent TN and TP concentrations may be attributed to the regulatory effect of temperature on microbial activity [23], as well as the addition of excess carbon sources during treatment in response to high TN and TP levels [20,24]. Moreover, the effluent TP concentration was significantly influenced by the influent water volume. Despite the presence of complete biological phosphorus removal systems in each plant, the instability of phosphorus removal capabilities necessitates the installation of chemical phosphorus removal systems to compensate, making the effluent TP particularly sensitive to variations in the influent water volume.

Furthermore, the correlation between influent SSs and effluent SSs suggested that high SS levels in the influent could pose challenges to the separation of solids during treatment, leading to higher SS levels in the effluent. This underscores the need for efficient solid removal processes, such as enhanced coagulation or advanced filtration technologies, to maintain effluent quality. The effluent SSs in each plant were significantly influenced by BOD_5_, COD_cr_, and TP. This was due to the degradation of BOD_5_ and COD_cr_, leading to the generation of more microbes and, subsequently, more Extracellular Polymeric Substances, which, in turn, affected the effluent SSs [25]. Additionally, chemical phosphorus removal can lead to changes in SSs.

Overall, the correlation analysis provides valuable insights into the interrelationships between various treatment parameters, offering guidance on where operational adjustments or technological upgrades may be most beneficial. For instance, plants experiencing strong correlations between the influent organic load and effluent quality could benefit from pretreatment enhancements, while those with considerable temperature-related impacts on nutrient removal could consider implementing seasonal process adjustments or supplementary heating strategies.

## 4. Conclusions

This study performed a comprehensive analysis of the operational performance of three WWTPs in Handan City, focusing on the relationships between influent and effluent parameters over a three-year period. The findings emphasize the crucial role of various factors, such as temperature, C/TN and C/TP ratios, and influent pollutant concentrations, in determining the efficiency of nutrient removal processes, particularly under varying seasonal conditions.

The analysis results showed that the Level A standard for effluent BOD_5_ in Plants D, T, and X was 95.7%, 68.4%, and 69.2%, the Level A standard for effluent COD_Cr_ in Plants D, T, and X was 100%, 97.5%, and 100%, the Level A standard for effluent NH_4_^+^-N in Plants D, T, and X was 96.2%, 84.6%, and 78.4%, and the Level A standard for effluent TN in Plants D, T, and X was 95.3%, 79.2%, and 76.1%. The correlation results showed that there was a strong positive correlation between influent BOD_5_ and COD_Cr_ concentration, a strong correlation between effluent TN concentration and effluent NH_4_^+^-N concentration, and a negative correlation between influent temperature and effluent TP concentration.

The analysis revealed that optimizing operational parameters, including nitrification and denitrification processes, is essential for ensuring stable TN and TP removal, particularly during colder months when microbial activity is reduced. Furthermore, this study underscores the importance of integrating both biological and chemical phosphorus removal systems to ensure consistent effluent quality, as biological phosphorus removal alone proved insufficient under certain conditions.

Additionally, this study highlights the necessity of advanced solid removal techniques, such as enhanced coagulation or filtration, to address the challenges posed by high SS levels in influent. The correlation analysis provided valuable insights into the interdependencies of treatment parameters, offering a basis for targeted operational adjustments and potential technological upgrades.

## Figures and Tables

**Table 1:** Characteristics of wastewater treatment plants in the main urban area of Handan City.

Name	Footprint	Population Equivalent	Design Capacity	Actual Capacity of Operation	Range of Water Collection
WWTPs D	270 acres	350,000 p.e.	160,000 m^3^/day	60,000 m^3^/d	East of the railroad, south of Qin River, west of East Ring Road, and north of South Ring Road.
WWTPs T	81 acres	380,000 p.e.	100,000 m^3^/day	60,000 m^3^/d	East of Busan River, west of East Ring Road, south of North Ring Road, and north of Renmin Road.
WWTPs X	150 acres	450,000 p.e.	200,000 m^3^/day	85,000 m^3^/d	West of the Beijing–Guangzhou railway, within Outer Ring Road and east of the Beijing–Guangzhou railway, west of Busan River, north of Qin River, and south of North Ring Road.

## Data Availability

The original contributions presented in this study are included in the article; further inquiries can be directed to the corresponding authors.

## References

[1] Deng G., Yao X., Jiang H., Cao Y., He C. (2020). Study on the Ecological Operation and Watershed Management of Urban Rivers in Northern China. Water.

[2] Cremades R., Sanchez-Plaza A., Hewitt R.J., Mitter H., Tudose N.C. (2021). Guiding cities under increased droughts: The limits to sustainable urban futures. Ecol. Econ..

[3] Leeuwen K.V., Hofman J., Driessen P., Frijns J. (2019). The challenges of water management and governance in cities. Water.

[4] Koop S.H.A., Grison C., Eisenreich S.J., Hofman J., van Leeuwen K. (2022). Integrated water resources management in cities in the world: Global solutions. Sustain. Cities Soc..

[5] Li X., Li X., Li Y. (2022). Research on reclaimed water from the past to the future: A review. Environ. Dev. Sustain..

[6] Asiwal R.S., Sar S.K., Singh S., Sahu M. (2016). Wastewater Treatment by Effluent Treatment Plants. Int. J. Civ. Eng..

[7] Jin Z., Zhang X., Li J., Yang F., Kong D., Wei R., Huang K., Zhou B. (2017). Impact of wastewater treatment plant effluent on an urban river. J. Freshw. Ecol..

[8] Wang J., Yang H., Liu X., Chang J. (2020). The impact of temperature and dissolved oxygen (DO) on the partial nitrification of immobilized fillers, and application in municipal wastewater. RSC Adv..

[9] Bayo J., López-Castellanos J., Puerta J. (2016). Operational and Environmental Conditions for Efficient Biological Nutrient Removal in an Urban Wastewater Treatment Plant. CLEAN Soil Air Water.

[10] Nazif S., Forouzanmehr F., Khatibi Y. (2023). Developing a practical model for the optimal operation of wastewater treatment plant considering influent characteristics. Environ. Sci. Pollut. Res. Int..

[11] Luostarinen S., Sanders W., Kujawa-Roeleveld K., Zeeman G. (2007). Effect of temperature on anaerobic treatment of black water in UASB-septic tank systems. Bioresour. Technol..

[12] Nam K., Heo S., Kim S., Yoo C. (2023). A multi-agent AI reinforcement-based digital multi-solution for optimal operation of a full-scale wastewater treatment plant under various influent conditions. J. Water Process Eng..

[13] Wang X., Dong Y., Yu S., Mu G., Qu H., Li Z., Bian D. (2022). Analysis of the Electricity Consumption in Municipal Wastewater Treatment Plants in Northeast China in Terms of Wastewater Characteristics. Int. J. Environ. Res. Public Health.

[14] Zhang J.Y., Yang M., Zhong H., Liu M.M., Sui Q.W., Zheng L.B., Tong J., Wei Y.S. (2018). Deciphering the factors influencing the discrepant fate of antibiotic resistance genes in sludge and water phases during municipal wastewater treatment. Bioresour. Technol..

[15] Li W.Y., Xu Y., Feng J. (2009). Treatment of Coking Wastewater by Using an Immobilized-Microbial-Cell Anaerobic-Aerobic System. Energy Sources.

[16] Shi Y., Liu T., Quan X., Chen S., Yu H., Quan W. (2023). Enhanced nitrogen removal in the upgrading of municipal wastewater treatment plants by using zero-valent iron-modified biofilm carriers and clinoptilolite-modified biofilm carriers. Chem. Eng. J..

[17] Arcila J.S., Buitrón G. (2017). Influence of solar irradiance levels on the formation of microalgae-bacteria aggregates for municipal wastewater treatment. Algal Res..

[18] Qin G., Zou K., He F., Shao J., Zuo B., Liu J., Liu R., Yang B., Zhao G. (2023). Simultaneous determination of volatile phenol, cyanide, anionic surfactant, and ammonia nitrogen in drinking water by a continuous flow analyzer. Sci. Rep..

[19] Izadi P., Izadi P., Eldyasti A. (2020). Design, operation and technology configurations for enhanced biological phosphorus removal (EBPR) process: A review. Rev. Environ. Sci. Bio/Technol..

[20] Lu L., Guest J.S., Peters C.A., Zhu X., Rau G.H., Ren Z.J. (2018). Wastewater treatment for carbon capture and utilization. Nat. Sustain..

[21] Wang D., Li Y., Cope H.A., Li X., He P., Liu C., Li G., Rahman S.M., Tooker N.B., Bott C.B. (2021). Intracellular polyphosphate length characterization in polyphosphate accumulating microorganisms (PAOs): Implications in PAO phenotypic diversity and enhanced biological phosphorus removal performance. Water Res..

[22] Cota W., Soriano-Paos D., Arenas A., Ferreira S.C., Gómez-Gardees J. (2021). Infectious disease dynamics in metapopulations with heterogeneous transmission and recurrent mobility. New J. Phys..

[23] Krieger A.G., Zhang J., Lin X.N. (2021). Temperature regulation as a tool to program synthetic microbial community composition. Biotechnol. Bioeng..

[24] Soroosh H., Otterpohl R., Hanelt D. (2023). Influence of supplementary carbon on reducing the hydraulic retention time in microalgae-bacteria (MaB) treatment of municipal wastewater. J. Water Process Eng..

[25] He Q., Wang H., Xu C., Zhang J., Zhang W., Zou Z., Yang K. (2016). Feasibility and optimization of wastewater treatment by chemically enhanced primary treatment (CEPT): A case study of Huangshi. Chem. Speciat. Bioavailab..

